# Comparative activity of dimethyl fumarate derivative IDMF in three models relevant to multiple sclerosis and psoriasis

**DOI:** 10.1002/2211-5463.13969

**Published:** 2025-01-17

**Authors:** Yulin He, Guiyi Gong, Geovani Quijas, Simon Ming‐Yuen Lee, Ratan K. Chaudhuri, Krzysztof Bojanowski

**Affiliations:** ^1^ Department of Food Science and Nutrition The Hong Kong Polytechnic University Hung Hom China; ^2^ Sunny BioDiscovery Inc. Santa Paula CA USA; ^3^ Research Centre for Chinese Medicine Innovation The Hong Kong Polytechnic University Hung Hom China; ^4^ PolyU‐BGI Joint Research Centre for Genomics and Synthetic Biology in Global Ocean Resources The Hong Kong Polytechnic University Hung Hom China; ^5^ Sytheon (a Hallstar Company) Parsippany NJ USA

**Keywords:** fumarate, IDMF, Jurkat, microglia, multiple sclerosis, psoriasis, zebrafish

## Abstract

Dimethyl fumarate (DMF) is an anti‐inflammatory and immunoregulatory medication used to treat multiple sclerosis (MS) and psoriasis. Its skin sensitization property precludes its topical use, which is unfortunate for the treatment of psoriasis. Isosorbide di‐(methyl fumarate) (IDMF), a novel derivative of DMF, was synthesized to circumvent this adverse reaction and unlock the potential of topical delivery, which could be useful for treating psoriasis in the subpopulation of psoriatic MS patients, as well as in the general population. Here, we compared its therapeutic potential of this non‐sensitizing derivative with DMF and its therapeutic version Diroximel in three skin‐ and neuroinflammation models: the lck‐GFP zebrafish, activated BV‐2 murine microglia and human T‐lymphocyte Jurkat cell line. The results provide a comparative evaluation of the bioactivity of these three related chemical entities in models relevant to skin and neuroinflammation and expose several therapeutic advantages unique to IDMF.

## Graphical abstract

IDMF is a derivative of dimethyl fumarate (DMF) with a unique therapeutic potential at the intersection of psoriasis and multiple sclerosis. Here, we demonstrate that it compares favorably with DMF and its other derivatives, such as Diroximel, in the zebrafish, microglia, and lymphocyte models.

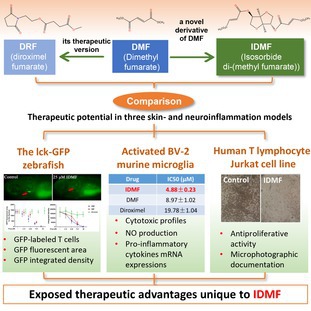

**Abbreviations**
DMF: dimethyl fumarate
DRF: diroximel fumarate
IDMF: isosorbide di‐(methyl fumarate)
MMS: monomethyl fumarate
MS: multiple sclerosis
*n* value: number of biologically‐independent replicates

Dimethyl fumarate (DMF) is an ester of fumaric acid used in human medicine to treat autoimmune diseases, such as multiple sclerosis (MS) and psoriasis [1]. Although the effectiveness of DMF in the treatment of relapsing–remitting MS and moderate to severe psoriasis is well established, gastrointestinal (GI) irritation limits compliance with oral intake, and skin sensitization prohibits its topical use altogether [2, 3, 4, 5]. And yet, the lack of serious systemic side effects makes this therapeutic modality valuable enough to pursue the search for improved dosages, derivatives and formulations with fewer adverse outcomes [6, 7, 8]. One such derivative is diroximel fumarate (DRF), which undergoes esterase cleavage in the GI tract to monomethyl fumarate (MMF), 2‐hydroxyethyl succinimide, RDC‐8439, and methanol, with MMF believed to be the active metabolite [9]. Although DRF saw improved compliance, adverse effects were found to persist [a 96‐week study (EVOLVE‐MS‐1) showed that 32% of patients given DRF still developed adverse GI effects] [10]. On the positive side, another study showed that the number of days with greater than mild GI symptoms was 46% lower in patients given DRF 462 mg BID as compared to DMF 240 mg BID [11].

The increased therapeutic index of DRF may be due to the improved chemical formula of this fumarate, which results in its over 90% metabolization to HES and MMF, with only minor (< 10%) yield of RDC‐8439 and methanol [9]. Importantly, high methanol concentrations in the small intestine and skin, correlated with undesirable GI effects and skin sensitization observed with DMF and DRF treatment [12, 13].

Despite the improvement over DMF, DRF is still not suitable for topical application, and thus the need for a non‐sensitizing fumarate derivative remains unfulfilled. To fill this gap, we have developed a novel fumarate entity, isosorbide di‐(methyl fumarate) (IDMF), consisting of two methyl fumarate groups bound to a central isosorbide moiety [14, 15]. Like DMF, upon introduction to cell cultures, IDMF is converted to MMF (primarily by carboxylesterase 2). However, IDMF is projected to be less reactive than DMF due to its chemical structure and higher activation free energy barrier [14]. Accordingly, topical application does not appear to cause irritation or sensitization in a cellular or several animal models (i.e., rat, rabbit, guinea pig) [14].

Two previous studies with IDMF tested by itself and in comparison with other fumarates demonstrated a greater decrease in the expression of genes associated with the proliferative reactive phenotype in human primary astrocytes, as well as inhibition of NF‐κB target genes [14, 15, 16]. The results of those studies provided the underlying hypothesis that IDMF may have a better therapeutic index than the older fumarates and warrant further research. Because DMF is known to reduce inflammation via its inhibitory effect on microglia [17] and T cells [18], here, we extended those comparative studies to the LPS‐activated BV‐2 murine microglial model, as well as the zebrafish T‐lymphocyte and human Jurkat cell models relevant to neurotoxicity and skin inflammatory diseases. The overreaching goal of this study was to make a preliminary assessment of the therapeutic potential of IDMF as compared to DMF and DRF in these three preclinical models.

## Materials and methods

### Reagents and chemicals

All reagents and chemicals were from Sigma (St. Louis, MO, USA), except if stated otherwise. DRF (cat.# 29111) was from Cayman Chemicals (Ann Arbor, MI, USA).

### Synthesis of isosorbide di‐(methylfumarate) (IDMF)

To a suspension of isosorbide (50 g, 0.34 mol) in ethyl acetate (500 mL) was added MMF (97.5 g, 0.75 mol) followed by 4‐dimethylaminopyridine (DMAP) (8.3 g, 0.06 mol). The reaction mixture was cooled to 0 °C (ice bath) and a solution of dicyclohexylcarbodiimide (DCC) (168 g, 0.75 mol) in ethyl acetate (800 mL) was added drop wise. The reaction mixture was then allowed to warm up to room temperature and stirred for 16 hrs. Precipitates were filtered and washed with Ethyl acetate (300 mL). The filtrate was concentrated under vacuum to give 176 g crude product. This crude product was shaken in hexane (1 L), filtered and the solid again washed with hexane (500 mL). The resultant solid product was dried and subsequently purified on a short path of silica gel using ethyl acetate (0–50%)/hexane (100–50%) gradient to give 87 g of white solid. This solid was recrystallized in hot ethyl acetate (500 mL) to give a total of 60 g of pure IDMF, mp 106–108 °C, with a purity of about 98.5% as determined by HPLC. Structure of IDMF was established by 1HNMR, 13CNMR and MS analyses.

### Lck‐GFP transgenic zebrafish model

A transgenic zebrafish model expressing green fluorescent protein (GFP) under the control of the T‐lymphocyte‐specific tyrosine kinase (lck) promoter was engineered using GFP transgene under the lck promoter cloned into a pBluescript vector as described before [19]. Tg(lck:GFP) zebrafish were obtained from the Institute of Chinese Medical Sciences (ICMS) and maintained as described previously [20]. Briefly, adult animals were maintained at 28.5 °C with a 14 h:10 h light/dark photoperiod. The embryos were raised in embryo medium (0.54 mm KCl, 13.7 mm NaCl, 0.044 mm KH_2_PO_4_, 0.025 mm Na_2_HPO_4_, 0.1 mm MgSO_4_, 0.13 mm CaCl_2_, and 42 μm NaHCO_3_; PH 7.4) at a temperature of 28.5 °C. Ethical approval (approval no: UMARE‐021b‐2020) for the animal experiments was granted by the Animal Research Ethics Committee, University of Macau. Five‐day‐old lck‐GFP transgenic embryos were treated with dexamethasone (100 μg·mL^−1^) or with indicated concentrations of DMF, IDMF and DRF in egg water. Fish were analyzed at 3 days posttreatment for T cell ablation as detected by loss of GFP fluorescence in the thymus.

### Cellular models

The murine BV‐2 microglial cell line was obtained from the China Center for Type Culture Collection (Wuhan, China) and grown at 37 °C/5% CO_2_ in DMEM supplemented with 10% FBS, 1% penicillin–streptomycin. BV‐2 cells were treated with the indicated concentrations of DMF, IDMF and diroximel, with or without LPS (500 ng·mL^−1^, added 1 h after the fumarates) for the indicated times. After that, cells were incubated with Cell Counting Kit‐8 (CCK‐8, Beyotime Biotechnology, Shanghai, China). The absorbance at 450 nm was measured using a FlexStation 3 Multi‐Mode Microplate Reader (Molecular Devices, Sunnyvale, CA, USA) and cell viability was expressed as a percentage of vehicle control. Microglial production of NO in the culture media supernatants was assessed using a Nitrite Concentration Assay Kit (Beyotime Biotechnology) according to the manufacturer's protocol. Absorbance was determined at 540 nm.

Suspensions of Jurkat cells (Clone E6‐1, cat.# TIB‐152, ATCC, Manassas, VA) were cultured at 37 °C/5% CO_2_ in RPMI‐1640 medium (cat.# 30–2001, ATCC) until subconfluence and were exposed to the test materials for 72 h. The test substances (MMF, DMF, DRF, and IDMF) were added in at least three replicates to the cell cultures in a 96‐well plate. Cell culture metabolic activity was measured by the resazurin mitochondrial reductase assay [21]. This assay converts the nonfluorescent blue dye resazurin to the fluorescent red dye resorufin. Mechanistically, resazurin is taken up by metabolically active cells, which maintain a reducing cytosolic environment and thus reduce the absorbed resazurin to the highly fluorescent product resorufin, which is secreted into the extracellular medium. The fluorescence emitted by resorufin was measured with a multimode SpectraMax i3x platform (Molecular Devices) at 560 nm/590 nm. Microscopic documentation of the cell cultures was performed with an EVOS 5000 imaging system (ThermoFisher Scientific, Waltham, MA, USA).

### Quantitative PCR (qPCR) assay

Total RNA of BV‐2 cells was extracted using the High Pure RNA Isolation Kit (Tiangen Biotech, Beijing, China). Isolated RNA was then reverse‐transcribed into cDNA using the Evo M‐MLV RT Premix for qPCR Kit (Accurate Biology, Shandong, China). The qPCR assay was conducted using SYBR Green Premix Pro Taq HS qPCR Kit (Accurate Biology) with the QuantStudio 7 Flex Real‐Time PCR System (ThermoFisher). The cycling conditions were as follows: 50 °C for 2 min and 95 °C for 10 min, followed by 40 cycles of 95 °C for 15 s and 60 °C for 30 s. The relative expression of mRNA was calculated after normalization to glyceraldehyde 3‐phosphate dehydrogenase (GAPDH). The primer sequences used are listed in Table 1.

**Table 1:** Primers used for quantitative RT‐PCR.

Primer name	Sequence
*iNOS* forward	5′‐CAAGAGTTTGACCAGAGGACC‐3′
*iNOS* reverse	5′‐TGGAACCACTCGTACTTGGGA‐3′
*COX2* forward	5′‐TTGAAGACCAGGAGTACAGC‐3′
*COX2* reverse	5′‐GGTACAGTTCCATGACATCG‐3′
*IL‐1β* forward	5′‐GGCAACTGTTCCTGAACTCAACTG‐3′
*IL‐1β* reverse	5′‐CCATTGAGGTGGAGAGCTTTCAGC‐3′
*IL‐6* forward	5′‐CCACTTCACAAGTCGGAGGCTT‐3′
*IL‐6* reverse	5′‐CCAGCTTATCTGTTAGGAGA‐3′
*TNF‐α* forward	5′‐CCTATGTCTCAGCCTCTTCT‐3′
*TNF‐α* reverse	5′‐CCTGGTATGAGATAGCAAAT‐3′
*GAPDH* forward	5′‐ATGTACGTAGCCATCCAGGC‐3′
*GAPDH* reverse	5′‐AGGAAGGAAGGCTGGAAGAG‐3′

### Statistical significance

*P* values representing statistical significance were calculated using two‐tailed *t*‐test and the threshold of statistical significance was fixed at *P* = 0.05 and 15% difference compared to the water control group.

## Results

### Murine BV‐2 microglial model

**Figure 1:**
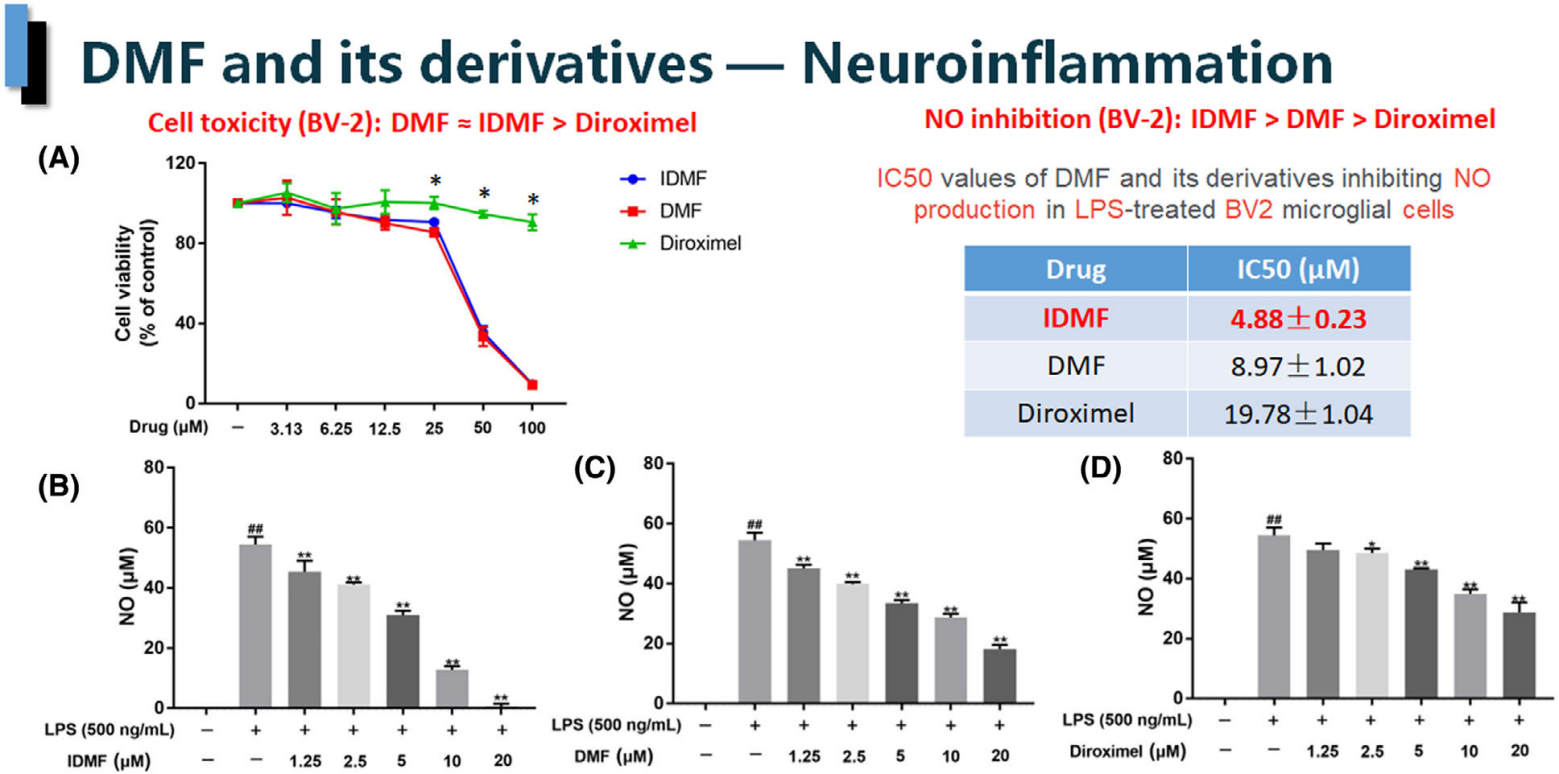
Effect of DMF and its derivatives on the viability and nitric oxide (NO) output in the BV‐2 microglia cell model. (A) Cell viability examined by the CCK‐8 assay, showing identical cytotoxic profiles of DMF and IDMF, and lack of cytotoxicity up to 100 μm for DRF (*P < 0.05 Diroximel vs. IDMF and DMF). (B–D) IDMF (B) inhibits the production of nitric oxide (NO) significantly better than DRF (D) at 2.5 μm and higher doses (*P* < 0.05). Furthermore, IDMF inhibits the production of NO significantly better than DMF (C) at 5 μm and up. ##*P* < 0.05 LPS‐stimulated vs. non‐stimulated controls; **P < 0.05 vs. LPS‐stimulated control. Error bars indicate standard deviations. *N* value: 3. *P* values were calculated using two‐tailed *t*‐test.

Activated microglia are key pathological effectors of neuroinflammation in the central nervous system (CNS) [22]. DMF is known to inhibit iNOS in the BV‐2 microglial cell line [23]; therefore, we chose this model to compare the anti‐neuroinflammatory potential of IDMF against the two other fumarates. Figure 1 shows that DMF and IDMF inhibited the proliferation of LPS‐activated BV‐2 cells with the same efficiency, both having the same 50% inhibitory concentration (IC_50_) of 30 μm. In contrast, DRF had no statistically significant effect up to the highest tested dose (100 μm).

DMF was also the weakest inhibitor of nitric oxide (NO) secretion by microglia, while IDMF had the best activity, achieving more than 50% greater inhibition than DMF and fivefold greater activity, in terms of IC_50_, than DRF (Fig. 1B). The difference in NO inhibition between IDMF and the two other fumarates was statistically significant.

**Figure 2:**
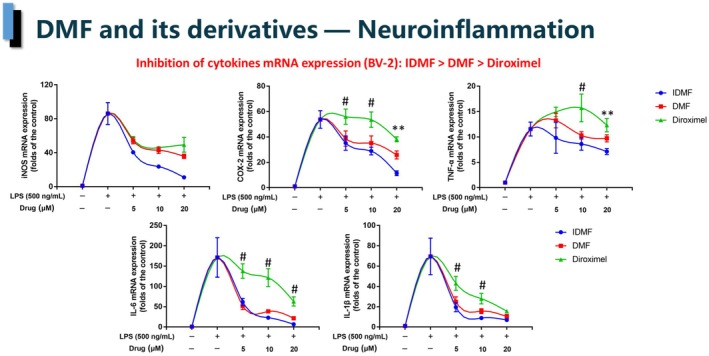
DMF and its derivatives inhibit the expression of iNOS, COX‐2, TNF‐α, IL‐1β and IL‐6 in LPS‐stimulated BV‐2 cells unequally, as determined by RT‐PCR. *P < 0.05 IDMF vs. DMF and Diroximel; #*P* < 0.05 IDMF and DMF vs. Diroximel; **P < 0.05 IDMF vs. DMF and Diroximel, and DMF vs. Diroximel. Error bars indicate standard deviations. *N* value: 3. *P* values were calculated using two‐tailed *t*‐test.

In order to determine whether the decrease in NO secretion observed with the tested fumarates was due to transcriptional regulation, the expression of the iNOS gene (NOS2) was measured by qPCR. The results revealed a correlation between the expression of NOS2 and NO secretion, with IDMF again being the most efficient inhibitor (Fig. 2). qPCR was then used to quantify four other markers of neuroinflammation—COX‐2, TNF‐α, IL‐6, and IL‐1β. The expression of these four genes was best inhibited by IDMF, followed closely by DMF, while, again, DRF was the weakest inhibitor.

Besides the anti‐neuroinflammation potential due to the subversion of microglia, DMF is known to reduce inflammation via the inhibitory action on T cells [18]. Therefore, we compared the ability of the three fumarates to exert an antiproliferative effect on GFP‐labeled T cells in the zebrafish model, as well as in the human T‐lymphocyte Jurkat cell line.

### Lck‐GFP transgenic zebrafish model

**Figure 3:**
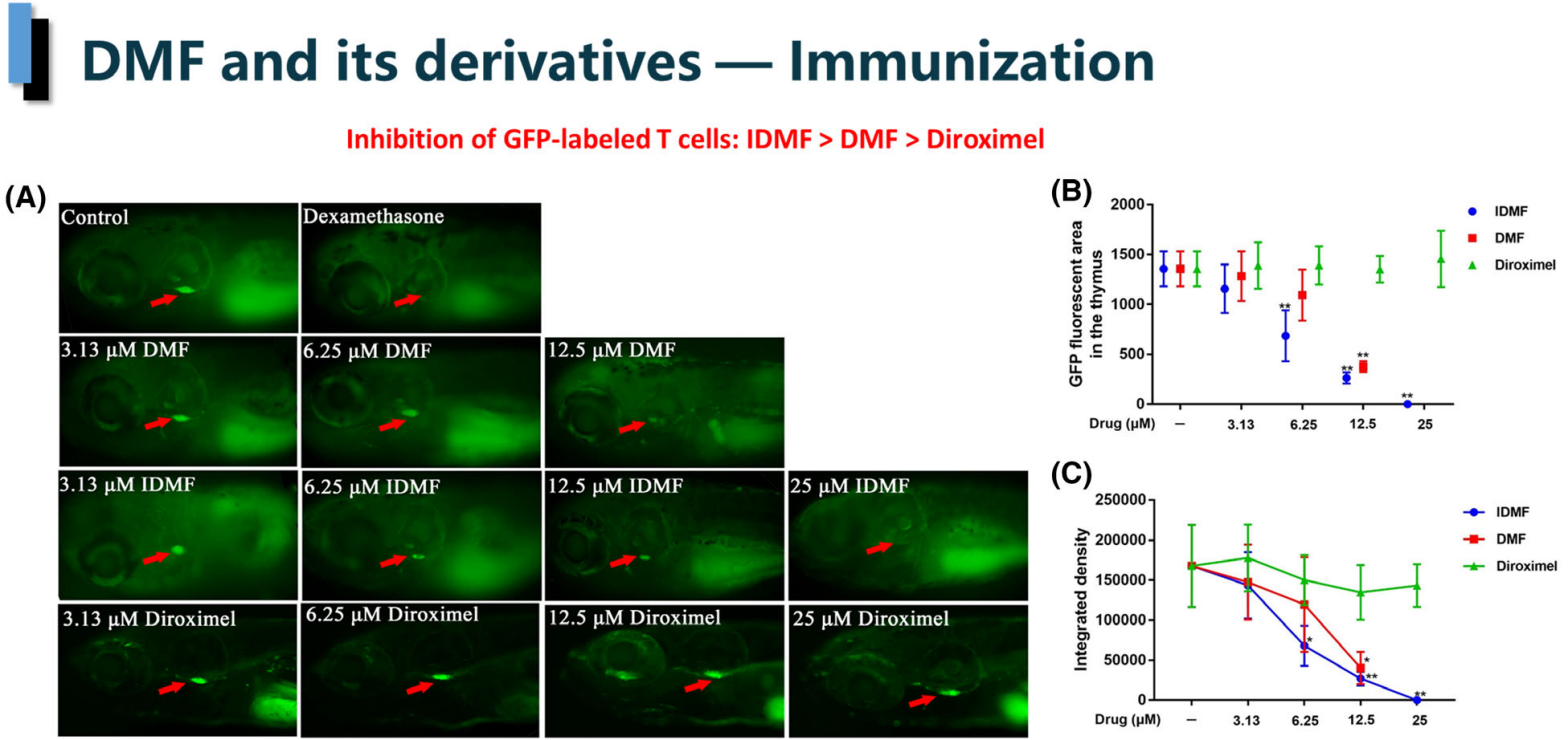
Effect of DMF and its derivatives on thymic T cell maturation in the lck‐GFP transgenic zebrafish model. (A) GFP‐labeled T cells in 8‐day‐old lck‐GFP zebrafish are ablated in response to DMF and its derivatives. Arrowheads denote GFP‐labeled cells in the thymus. (B) The GFP fluorescent area in the thymus of lck‐GFP zebrafish. (C) The integrated density of the GFP fluorescence. *P < 0.05 vs. untreated control; **P < 0.01 vs. untreated control. Error bars indicate standard deviations. Scale bar = 250 nm; *N* value: 15. *P* values were calculated using two‐tailed *t*‐test.

GFP‐labeled T cells in a zebrafish model closely replicate the maturation of T cells in the human thymus [19]. The results presented in Fig. 3 show that DMF and IDMF strongly ablated GFP‐labeled T lymphocytes in the thymus (IC_50_ 9.58 ± 0.39 μm and 6.35 ± 1.14 μm respectively), while DRF did not have any statistically significant effect. The positive control dexamethasone at 100 μg·mL^−1^ resulted in complete suppression of the GFP signal in the thymus, providing technical validation of the experiment.

### Jurkat cell line

**Figure 4:**
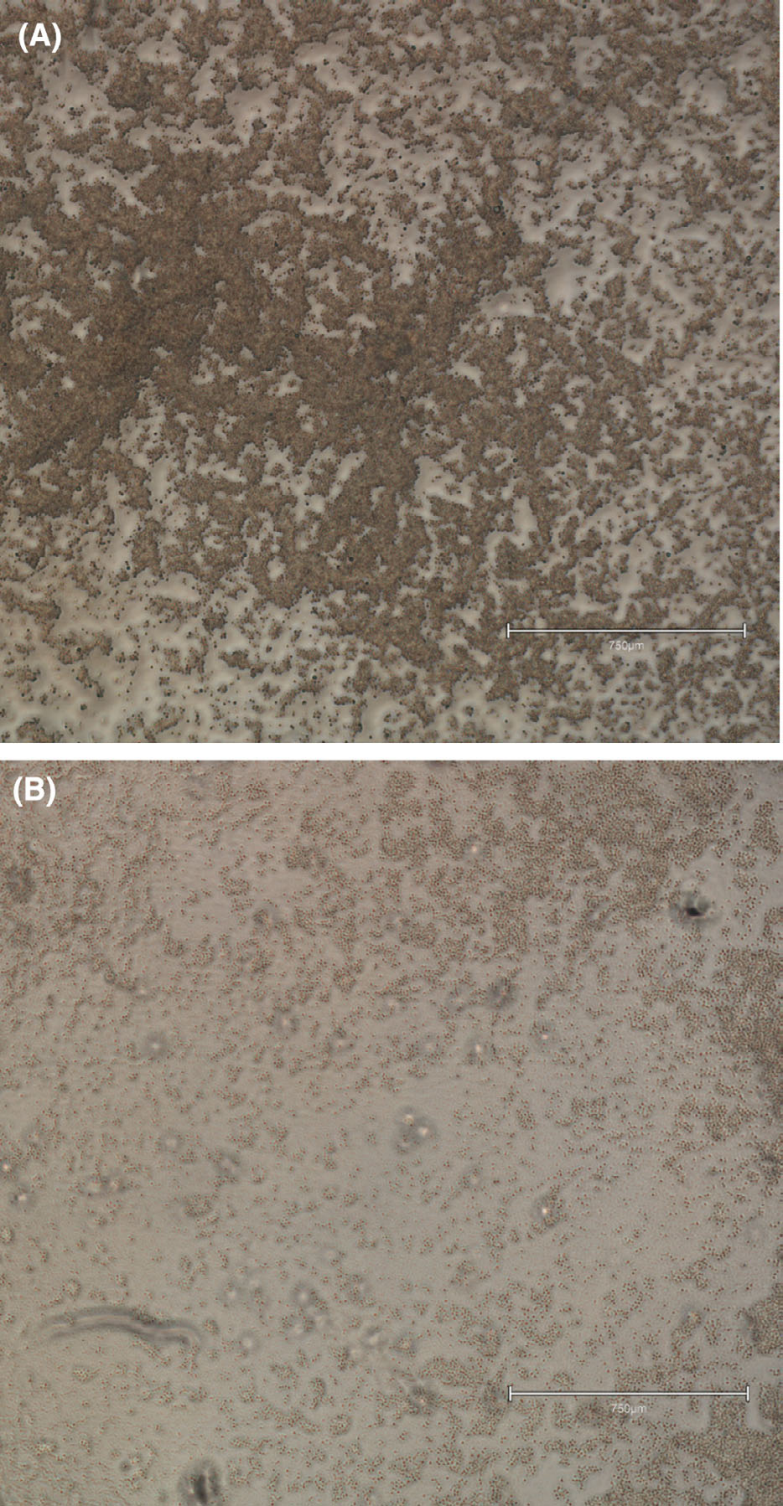
Microphotographic documentation of Jurkat cells cultured without (A) or with (B) IDMF (25 μg·mL^−1^). Initial magnification: ×4, bar: 750 μm. *N* value: 3.

To conclude with a human model, the Jurkat T‐lymphocyte cell line was incubated with the three fumarates, as well as their active metabolite MMF. Table 2 backed by Fig. 4 shows that the antiproliferative activity in this experimental model was similar to that in BV‐2 cells, in terms that IDMF was the most active (IC_50_ 22 μm), followed by DMF (IC_50_ 65 μm) and DRF (IC_50_ 73 μm). MMF had the weakest activity, approaching 50% inhibition at 200 μm.

**Table 2:** Comparative effect of IDMF, DMF, MMF, and DRF on the metabolic activity in Jurkat cells measured by the resazurin assay and expressed as % of water‐treated control. *P* values were calculated using two‐tailed *t*‐test. DMF, dimethyl fumarate; DRF, diroximel fumarate; IDMF, isosorbide di‐(methyl fumarate); MMS, monomethyl fumarate; SEM, standard error of the mean.

Compound	Jurkat cell viability (% control)	*P* value	SEM
IDMF 25 μg·mL^−1^	18	0.000	1.5
IDMF 5 μg·mL^−1^	52	0.001	5.7
IDMF 1 μg·mL^−1^	83	0.220	6.2
DMF 25 μg·mL^−1^	28	0.000	6.0
DMF 5 μg·mL^−1^	57	0.014	7.2
DMF 1 μg·mL^−1^	84	0.238	3.1
MMF 25 μg·mL^−1^	53	0.005	8.2
MMF 5 μg·mL^−1^	76	0.160	8.9
MMF 1 μg·mL^−1^	113	0.239	12.4
DRF 25 μg·mL^−1^	22	0.000	8.9
DRF 5 μg·mL^−1^	110	0.473	4.1
DRF 1 μg·mL^−1^	84	0.256	3.5

## Discussion

Psoriasis and MS appear to share many pathological cues, such as the involvement of IL‐17 [24], often leading to clinical comorbidity [25, 26]. This comorbid condition presents an opportunity to use one medication with dual (oral and topical) delivery routes to treat both diseases. Fumarates present such therapeutic opportunity (DMF is FDA‐approved to treat MS and psoriasis *per os*); however, skin sensitization triggered by the existing chemical entities of this category precluded their topical use so far. In previous studies, we used multiple models (human dermal fibroblasts, keratinocytes, as well as rats, rabbits and guinea pigs) to evaluate the effects of IDMF in the therapeutic context of MS and psoriasis [14], notably showing a lack of skin sensitization in animals. Moreover, microarray profiling was performed to compare this new fumarate with DMF and DRF in a human astrocyte model [15, 16]. This functional comparison uncovered further unique effects of IDMF not replicated by other fumarates. Here, we extended this evaluation to three other models of inflammation relevant to MS and psoriasis—LPS‐treated murine microglial BV‐2 cells, Ick‐GFP zebrafish, and the human Jurkat cell line.

Microglia are the resident macrophages of the central nervous system. Their proliferative activation (such as in the LPS model used herein) contributes to the progression of the disease [27, 28, 29, 30], and its inhibition may be beneficial in the treatment course of MS [31]. Here, we found that DMF and IDMF had comparable dose‐dependent inhibitory effects on LPS‐stimulated BV‐2 microglial proliferation, while DRF had no statistically significant effect (Fig. 1A). Further studies need to be performed to tease out this effect from the inhibition of cell viability, which would indicate toxicity.

Nitric oxide (NO) and cytokine secretion by pathologically activated microglia is believed to play an important role in the progression of many neurodegenerative diseases, including MS [32]. We therefore undertook a comparative analysis of the effects of DMF, DRF and IDMF on these markers of neuroinflammation in LPS‐challenged BV‐2 cells using low doses, which did not cause a decrease in cell proliferation. The results (Figs 1B–D and 2) show that IDMF consistently overperformed DMF and DRF in its ability to inhibit the secretion of NO and the expression of genes encoding the cytokines COX‐2, IL‐1β, IL‐6 and TNF‐α by LPS‐activated microglial cells. Furthermore, we identified the downregulation of NOS2 gene expression as the probable mechanistic cause of decreased NO secretion. Interestingly, the strength of this downregulation matched the potency to inhibit NO production for each of the tested fumarates. Better cytokine‐inhibitory activity of IDMF in microglia corroborates with results obtained in normal human astrocytes, where IDMF, but not DRF or MMF, significantly decreased the expression of IL‐6 (by 90%) [16]. IL‐6 has been strongly associated with pathogenicity in MS and psoriasis, and is considered to be an attractive potential therapeutic target for both diseases [33]. Although concerns have been raised about the efficiency of the anti‐IL‐6 signaling pathway‐specific antibody tocilizumab in psoriasis [34], this may reflect the shortcomings of the antibody itself, as in the case of anti‐IL‐17A treatment, where changing the therapeutic antibody, while maintaining the same target improved the outcome [35]. Furthermore, a decrease in the IL‐6 level is consistently associated with positive outcomes in clinical trials of both diseases [36, 37, 38].

Besides microglia control, strategies for the treatment of psoriasis and MS involve elimination of activated T cells [39, 40]. The Ick‐GFP zebrafish model allows to evaluate the sensitivity of thymic T cells to chemical ablation by therapeutic candidates, *in vivo*. Importantly, the sensitivity of this zebrafish model tends to parallel the mouse and human ones, as determined, for example, by its sensitivity to dexamethasone [41, 42], a corticosteroid medication used in treatment of both diseases [45, 46]. It also bears therapeutic significance in MS, as underscored by the finding that the removal of the thymus from newborn rats prevented the development of experimental autoimmune encephalomyelitis (EAE) later in life [43] and that thymic hyperplasia is overrepresented in patients with multiple sclerosis [47]. Finally, lck plays a key role in T‐cell activation and has been considered as a druggable target for multiple sclerosis [48].

The comparison of the effects of the three fumarates on the thymic T cell ablation in the lck‐GFP zebrafish model shows that DRF was ineffective, while DMF and IDMF demonstrated a dose‐dependent activity, with IDMF being the best overall performer. The inhibition obtained with DMF and IDMF was on par with the positive control dexamethasone (Fig. 3). This result underscores the mechanistic difference between DRF and DMF. It also indicates that IDMF is a closer functional relative of DMF than DRF is.

In order to corroborate these zebrafish data with a human model, IDMF, DMF and DRF were tested on the Jurkat T‐lymphocyte cell line. Furthermore, the DMF active metabolite MMF, was added to this experimental design. Jurkat cells are a relevant research tool to both MS and psoriasis [44]. The results obtained in this model reflected the hierarchy observed in the animal thymus, with IDMF having the lowest IC_50_, followed by DMF and DRF. The activity of MMF was too low to reach 50% inhibition, which hovers above 200 μm for that compound. Together, these findings indicate that among the tested fumarates, IDMF is the most active proliferation inhibitor of Jurkat cells.

Overall, these results show that at the highest non‐toxic dose (as determined in the activated BV‐2 microglia model), the therapeutic effectiveness of DMF and IDMF is considerably stronger than DRF's. Notwithstanding that increasing DRF concentrations may remediate these differences—clinical trials show that the optimal dose of DRF is double of DMF [11]—IDMF has the potential to deliver DMF's level of effectiveness with lesser adverse reactions than DMF, resulting in a truly improved therapeutic index.

## Conflict of interest

The authors declare that there is no conflict of interest regarding the publication of this paper. None of the Authors is involved in the commercialization of any fumarates reported in this paper and IDMF is not currently being commercialized.

### Peer review

The peer review history for this article is available at https://www.webofscience.com/api/gateway/wos/peer‐review/10.1002/2211‐5463.13969.

## Author contributions

RKC, SM‐YL, and KB conceived and designed the project, YH, GG, and GQ acquired the data, YH, GG, SM‐YL, and KB analyzed and interpreted the data, KB wrote the paper.

## Data Availability

Data are available on request. The request should be addressed to the corresponding author (Dr Krzysztof Bojanowski) by email (kbojanowski@sunnybiodiscovery.com) or telephone (+1 805 229 7580).
